# Vascular dementia: Diagnostic criteria and supplementary exams. Recommendations of the Scientific Department of Cognitive Neurology and Aging of the Brazilian Academy of Neurology. Part I

**DOI:** 10.1590/S1980-57642011DN05040003

**Published:** 2011

**Authors:** Eliasz Engelhardt, Carla Tocquer, Charles André, Denise Madeira Moreira, Ivan Hideyo Okamoto, José Luiz de Sá Cavalcanti, Working Group on Alzheimer’s Disease and Vascular Dementia of the Brazilian Academy of Neurology

**Affiliations:** 1 Full Professor (retired) – UFRJ, Coordinator of the Cognitive Neurology and Behavior Sector, INDC, CDA/IPUB, UFRJ, Rio de Janeiro RJ, Brazil; 2 Neurologist, Masters and PhD in Neuropsychology, Claude Bernard University, France; 3 Associate Professor of Neurology, Faculty of Medicine, UFRJ. Medical Director of SINAPSE Rehabilitation and Neurophysiology, Rio de Janeiro RJ, Brazil; 4 Adjunct Professor of Radiology, School of Medicine, UFRJ. Head of Radiology Sector, INDC, UFRJ, Rio de Janeiro RJ, Brazil; 5 Department of Neurology Neurosurgery, UNIFESP, Institute of Memory, UNIFESP, São Paulo SP, Brazil; 6 Adjunct Professor of Neurology, INDC, UFRJ. Cognitive Neurology and Behavior Sector, INDC, UFRJ, Rio de Janeiro RJ, Brazil

**Keywords:** recommendations, vascular dementia, criteria, neuroimaging, laboratory exams

## Abstract

Vascular dementia (VaD) is the most prevalent form of secondary dementia and the second most common of all dementias. The present paper aims to define guidelines on the basic principles for treating patients with suspected VaD (and vascular cognitive impairment - no dementia) using an evidence-based, systematized approach. The knowledge used to define these guidelines was retrieved from searches of several databases (Medline, Scielo, Lilacs) containing scientific articles, systematic reviews, meta-analyses, largely published within the last 15 years or earlier when pertinent. Information retrieved and selected for relevance was used to analyze diagnostic criteria and to propose a diagnostic system encompassing diagnostic criteria, anamnesis, as well as supplementary and clinical exams (neuroimaging and laboratory). Wherever possible, instruments were selected that had versions previously adapted and validated for use in Brazil that take into account both schooling and age. This task led to proposed protocols for supplementary exams based on degree of priority, for application in clinical practice and research settings.

## Abstract (pt)

A demência vascular (DV) é a forma mais prevalente de demência secundária e asegunda forma mais comum. O presente artigo visa definir recomendações dos princípios básicos para tratamento dos pacientes com suspeita de DV (e comprometimento cognitivo leve sem demência) usando uma abordagem sistematizada baseada em evidências. O conhecimento usado para definir estas recomendações foi recuperado de pesquisa de várias bases de dados (Medline, Scielo, Lilacs) contendo artigos científicos, revisões sistemáticas, meta-análises, publicados nos últimos 15 anos, ou antes, se pertinente. As informações recuperadas e selecionadas pela relevância foram usadas para analisar os critérios diagnósticos e propor um sistema diagnóstico incluindo critérios diagnósticos, anamnese, bem como exames complementares (neuroimagem e laboratório). Sempre que possível, os instrumentos foram selecionados com versões previamente adaptadas e validadas para uso no Brasil, segundo escolaridade e idade. Os protocolos propostos para exames complementares basearam-se no grau de prioridade, para aplicação na prática clínica ou em pesquisa.

## Introduction

Vascular dementia (VaD) is characterized by cognitive impairment, functional decline, behavioral disorders and neurological symptoms secondary to cerebrovascular disease (CVD). Vascular cognitive impairment (VCI) includes from very mild forms of impairment (VCI no dementia [CIND] and vascular mild cognitive impairment [VMCI]) to more severe forms, including VaD and its clinical stages,^1-5^ thus constituting a VCI/VaD spectrum. CVD can manifest associated with AD, constituting mixed forms such as AD+CVD and Mixed Dementia (MD).^6-9^ Pure forms of VCI/VaD associated with AD constitute vascular cognitive disorder (VCD),^10^ a concept later incorporated into VCI.^11^

VaD (and likewise CIND) is a clinically and anatomically heterogeneous condition. The clinical characteristics of VaD differ to those of most neurodegenerative diseases, since the latter tend to typically present sequential and predictable progression according to the underlying pathology (e.g. AD [amnestic form]). This heterogeneity stems from pathophysiologic aspects such as:

- The presence of ischemic (or hemorrhagic) intracerebral lesions (vascular ictus, cerebral vascular accident [CVA], cerebrovascular attack) with varying neuropathologic and neuroimaging characteristics. Ischemic ictus can be found in sites of large caliber arteries (single, multiple, strategic infarcts) and of lesions in regions nourished by small caliber arteries and arterioles (minor infarcts [which may be strategic], lesion in bordering areas, lacunes, white matter lesion). Hemorrhagic ictus can also cause similar pictures.^12-15^
- The relevance of site, size and number of lesions. Lesions must affect associative and/or limbic/paralimbic regions ("strategic areas")^16^, with initial symptoms and evolution varying accordingly. Thus, VaD can present a range of different neuropsychological patterns, with a predominance of executive dysfunction (mainly subcortical type), "scattered" impairments (due to multiple lesions) and impaired memory (hippocampal, median prosencephalic, and thalamic lesion) among others^17^. The size (volume) of infarct should be substantial (~100 cm^3^) in pure VaD and smaller (~50 cm^3^) in AD+CVD. The majority of patients with intermediate volume lesions (50 to 100 cm^3^) present with cognitive impairment.^16,18-19^ Infarcts smaller than the minimum stipulated volume can cause VaD, denoting the concept of "strategic site", 19 later referred to as VaD due to "strategic infarct".^20^

Detailed descriptions of classifications and characteristics of the main subtypes of VaD can be found in a number of published sources.^13,15,21^

The goal of the working group involved in the module "Vascular Dementia: diagnostic criteria and supplementary exams" was to put forward basic guidelines based on evidence for diagnosing VaD. This is the first task of its kind undertaken on VaD in our milieu, having led to a preliminary publication of a version of these guidelines^22^.

The previously published version was revised and split into two parts:

- diagnostic criteria and supplementary exams (part I).
- cognitive, functional and behavioral assessment (part II).

This first part of the diagnostic module for VaD covers diagnostic criteria and anamnesis, in addition to clinical and supplementary exams.

## Methods

The guidelines (recommendations and suggestions) were based on publications retrieved from electronic databases (Medline, Scielo, Lilacs) and encompassed scientific articles, systematic reviews, meta-analyses, largely published within the last 15 years, or earlier when pertinent. Consensus and Studies on the theme or related subjects were also examined.^11,23-31^

### Classification of evidence and levels of recommendation

The scientific evidence for diagnostic assessment was evaluated according to pre-established levels of certainty (Classes I, II, III and IV) and recommendations were graded according to strength of evidence *(Level A, B or C).* Additionally, important clinical issues were addressed for which evidence is questionable (Practice Option) and, when no evidence was available, recommendations were made based on the experience and consensus of the task force under "*Good Practice Point*" ^32,25^ (Table 1).

**Table 1:** Classification of evidence for diagnostic measurement and levels of recommendation.^32,25^

**Classification of evidence**	
**Class I**	Prospective study involving a broad spectrum of individuals with the suspected condition (using gold standard for defining cases), where test has been applied in blinded manner, enabling assessment of appropriate diagnostically accurate tests.
**Class II**	Prospective study involving a limited spectrum of individuals with the suspected condition, or well-planned retrospective study in broad spectrum of individuals with confirmed condition (using gold standard), compared with broad spectrum of control subjects, where test has been applied in blinded manner, and enables measurement of appropriate diagnostically accurate tests.
**Class III**	Retrospective study in limited spectrum of individuals with the confirmed condition and control subjects, in which tests have been applied in blinded manner.
**Class IV**	Any design methodology in which test has not been applied in blinded mode or is drawn from evidence based exclusively on opinion of a specialist or on a descriptive case series (without controls).
**Levels of recommendation**	
**Level A [Standard]**	Requires at least one convincing Class I study or at least two convincing Class II studies.
**Level B [Norm]**	Requires at least one convincing Class II study or indisputable Class III evidence.
**Level C**	Requires at least two convincing Class III studies.
**Practice option**	Requires Class IV evidence.
**Good practice point**	Based on the experience and consensus of the task force after considering important clinical questions for which no evidence (as per above) is available.

These guidelines may not be applicable under some circumstances and decisions on whether to apply recommendations must be taken in light of the individual clinical presentation of the case and of the resources available.^32^

### Diagnostic steps

The diagnostic steps outlined below should be followed systematically to determine the diagnosis of VaD as accurately as possible.

### Diagnostic criteria

The diagnostic criteria for VaD include the official sets (CID-10-CDP^33^ and DSM-IV^34^) as well as those devised specifically for research (CADDTC,^35^ NINDS-AIREN,^36^ NINDS-AIREN modified).^37^ Ischemic scores are also routinely used, the most common of which is the Hachinski (HIS).^38^

These criteria are similar on several aspects while differ on others, such as the definition of dementia (the majority incorporate memory impairment of varying degrees), characterized by vascular lesions and evidence of deficit.^13^ Thus, classification of cases can differ depending on the criteria used, with sensitivity ranging from 32.5% to 91.6%. Therefore, the current criteria are not interchangeable, potentially identifying different patient groups labelled as VaD.^39-41^ However, within their respective limits, all of the criteria are able to identify patients with VaD,^39,40,42,43^ and relatively successfully distinguish the disease from "pure" AD, and less effectively from MD. Concerning research or clinically-controlled trials, in which false positive cases should be excluded (generally AD+CVD), only highly specific criteria tend to be employed (such as NINDS-AIREN and CADDTC).^39,44,40^

The diagnosis of subcortical VaD is challenging using current criteria. Memory impairment may not be an initial or marked symptom, where another cognitive domain (or more than one), such as executive dysfunction may be more severely affected. The proposed NINDS-AIREN modified for subcortical VaD^45^ remedies this weakness by focusing on executive dysfunction and the presence of high grade subcortical ischemic lesions (leukoaraiosis, lacunes).^37,46,47^

Class I cliniconeuropathological studies in possible and probable VaD diagnosed by NINDS-AIREN have shown low sensitivity and high specificity for probable VaD (43%/95%),^48^ and likewise for possible VaD (55%/84%) and probable VaD (20%/93%),^44^ with similar aspects evident for the other criteria sets^44^. The cited criteria are deemed to offer the best sensitivity/specificity profile.

The HIS is a clinical instrument with differentiated scores for AD, VaD (MID type) and MD.^38^ A meta-analysis study with neuropathological correlation revealed sensitivity/specificity of 89%/89.3% for distinguishing between AD and VaD (MID), proving capable of correctly classifying 83.8% of patients with VaD (MID). This was found to be frequently elevated in patients diagnosed applying the NINDS-AIREN or CADDTC^49^. The lack of cognitive and neuroimaging data render the HIS insufficient when used alone.^42,43^

The key elements that emerge from these criteria constitute a diagnostic triad of VaD^21^.

**Dementia syndrome**

- With memory and/or executive function impairment.

**Vascular cause**

- Post-ictus or subcortical ischemia.

**Adequate relationship**

- Temporal [immediate, subacute, insidious].
- Functional [lesion to structures of cognitive integration].

Based on the diagnostic criteria, a general definition of VaD can be derived and, considering the variations presented, its simplified unification can be formulated as shown below. The basic characteristic of VaD is cognitive impairment of multiple domains, with compromise often being non-uniform. For VaD, memory impairment is a requisite (in most criteria) together with one or more of the following signs and symptoms: aphasia, apraxia, agnosia and executive dysfunction. Deficits of the disease must have a major impact on occupational or habitual activities and be marked by significant decline compared to previous level of functioning. The presence of neurological signs and symptoms is also a requirement, in addition to laboratory evidence or neuroimaging findings indicative of CVD, deemed to be etiologically related to the condition. The condition should not manifest exclusively during the course of delirium or major psychiatric disorder^21^.

**Recommendations** - A diagnosis of VaD must be based on specific criteria, with NINDS-AIREN being the most frequently used in research settings *(Level A).* The HIS can be recommended although with some restrictions, given a lack of cognitive and neuroimaging data *(Level C).* The diagnostic triad of VaD can represent a brief option for diagnosis *(Good Practice Point).*

### Anamnesis

Anamnesis is fundamental and must include questions on all aspects related to a dementia condition of vascular cause, such as mode of onset, pattern of progression, prior history (CVA, revascularization), comorbidities (SAH, DL, DM, anemia, sleep and psychiatric disorders), habits (eating, life-style, tobacco and alcohol use), familial and educational history. Specific questions on cognition, activities of daily living and behavior are necessary. It is important to obtain a full list of drugs used and prescribed, as well as alternative medications.^21,24,50,51^

**Recommendations** - Anamnesis is fundamental and data must be supplemented by a companion that is as well informed as possible *(Level A)*.

### Physical and neurological exams

A general physical exam can disclose frequent comorbidities, particularly in elderly patients, that can rapidly worsen cognitive, functional and behavioral status. These comorbidities or complications can include depression, cardiovascular disease, infections, dehydration, collateral effects of medications, delirium, falls, incontinence, anorexia and obesity. There is a strong correlation between comorbidities and cognitive status in VaD (Class IV).^52^ Clinical neurovascular assessment (palpation, auscultation) is also part of a thorough exam (see "Vascular neuroimaging"). Neurological examination is necessary and the presence of neurological symptoms makes up part of the diagnostic criteria of VD^51,53^ (Class II).

**Recommendations** - All patients presenting with, or suspected of having dementia must be submitted to a general physical exam aimed at detecting comorbidities, in addition to a neurovascular exam *(Good Practice Point)*, as well as a neurological exam *(Level B)*.

### Supplementary exams

Supplementary exams play a key role in the diagnostic process of VaD (and of vascular CIND). In this regard, neuroimaging is fundamental, particularly MR, and also laboratory exams on blood, and should be incorporated into the routine. Other exams can be performed according to the case being analysed^54^, classified into obligatory, desirable and z, in line with specific diagnostic needs (Table 2).

**Table 2:** Supplementary exams and indication hierarchy (see text for original references, translations and validations).

Supplementary exams
Structural neuroimaging^(1)^ • Cranium CT or MRI • Hippocampus (visual assessment)(CT or MRI)^(2)^
Vascular neuroimaging^(3)^ • Duplex ultrasonography (carotids and vertebrals) • CT-Angiography or MR-Angiography (intracranial and extra cranial carotid and vertebral arteries [veins])
Functional neuroimaging^(3)^ • Structural MRI (^1^H MRS) • Perfusion (CT or MRI)
Isotopic neuroimaging^(3)^ • SPECT or PET-CT/MRI
Clinical electrophysiology^(3)^ • EEG
Laboratory exams (clinical pathology) • Blood exam (routine, risk factors)^(1)^ • CSF Exam^(3)^• Genetic exam^(3)^ • Others (enzymes, antibodies, biopsy)^(3)^

(1) obligatory;

(2) desirable;

(3) occasional.

### Neuroimaging

Neuroimaging plays a pivotal role in the diagnostic process of patients with suspected dementia or that present with VaD (and vascular CIND), providing not only structural but also functional information.^54-56^

### Structural neuroimaging

This can be obtained through computed tomography scans (CT) or magnetic resonance imaging (MRI). Long considered merely for "excluding" brain lesions as the cause of dementia (e.g. tumors, hematomas, hydrocephaly), these techniques now exercise an important role of "including" diagnoses (e.g. neurodegenerative diseases, cerebrovascular diseases), through evidencing aspects considered characteristic of certain dementia types (e.g. hippocampal atrophy, as a marker of AD, or infarcts and lesions in white matter, as characteristics of VaD).^57-59^ It should be noted that despite confirmation of CVD on neuroimaging, the method cannot reliably diagnose VaD.^60^ However, the absence of CVD on neuroimaging offers strong evidence against dementia of vascular etiology.^61^

MRI is the method of choice for reaching a diagnosis of VaD (and vascular CIND), given its high sensitivity and spatial resolution, and ability to provide a greater amount of reliable data. The preferred magnetic field intensity is 1.5T or greater,^62^ where 0.5T may be acceptable.

The basic sequences needed are diffusion (DWI), 3D-T1, T2, T2-FLAIR and GE-T2 (gradient echo).^56,63-65^ CT should be used when no MRI device is available and in special situations (pace-maker fitted, intracranial ferromagnetic metal clips, psychological grounds etc.).^22^ Both techniques, within the scope of their characteristics, are able to obtain information on anatomy and presence of vascular lesions (infarcts, lacunes, changes in white matter, hemorrhages) and other pathologies, providing information on quantitative (number and volume) and topographic (localization) aspects. Ischemic changes in white matter (hypodensities - hyperintensities)(leukoaraiosis) can be assessed using visual and automated scales.^15,64-70^ The presence of hippocampal atrophy can also be assessed (see "Hippocampal atrophy").

Structural neuroimaging must be performed as routine and a number of diagnostic criteria for VaD expressly require neuroimaging as a core item^71,56^, such as the NINDS-AIREN, in which the technique is essential for diagnosing probable VaD, where lack of the method leads to a default diagnosis of the "possible" category^6^. Moreover, the criteria specify which vascular territories are "relevant" for VaD. Use of NINDS-AIREN operating guidelines for classifying radiological aspects led to a significant increase in diagnostic reliability among professionals assessing images from 40% to 60% (Class II).

**Hippocampal atrophy** - The assessment of degree of atrophy can be carried out by CT or MRI (with the latter being more reliable) using the visual assessment volumetric measurements (manual or automated), with the latter deemed more accurate.^73-76^ Comparative studies have shown good correlation among these techniques.^77,78^ Visual scales can be employed in clinical practice as well as clinical research, particularly in cross-sectional studies.^76,79-81^ T2-MRI or FLAIR can be used to distinguish the nature of the hippocampal atrophy. High signal, although also seen in AD, is not characteristic of this condition, being more often found in hippocampal sclerosis. On the other hand, ischemic changes in these regions can be better distinguished on FLAIR.^82^

MRI studies have shown hippocampal atrophy through visual (VaD) or volumetric assessment (subcortical VaD), to a lesser degree compared to AD but to a greater extent than in normal controls, in cases of dementia with similar severity.^83-86^ The findings on neuroimaging were confirmed by neuropathological studies showing that hippocampal volume was lower in VaD than in normal controls, but greater compared with atrophy found in AD.^87-89^

**Diffusion tensor** - Diffusion tensor imaging (DTI) is a technique for assessing the integrity of white matter fibers using quantitative fractionated anisotropy (DTI-FA) and tractography (DTI-TR). DTI-FA is an important technique in considering the large extension of white matter, and has been previously applied in clinical practice.^90-95^ DTI-TR can visualize the bundles interconnecting various regions whose interruption can cause a range of different disconnection syndromes. The method is not routinely used in clinical practice.^95,96^

### Vascular neuroimaging

Neurovascular assessment entails, beyond clinical examination, duplex ultrasonography (USG) of the extracranial carotid and vertebral arteries and CT-angiography or MRI-angiography of intracranial and extracranial carotid and vertebral arteries (from their point of origin).^97,98^ USG tends to be the initial exam and enables visualization of the vascular wall, detecting atheromatous plaques and stenosis while also providing a measure of intima-media thickness of the carotids, an early marker for wall pathology and cardiovascular risk factor. The characteristics of blood flow can also be verified, constituting a functional aspect.^99^ MRI-angiography and CT-angiography visualizes the entire cervical and intracranial tree (and venous system when necessary) and the vascular pathology present (atheromatous plaques, stenosis) which is related to flow and perfusion disorders with potential to cause brain lesions.^97-102^

**Recommendations** - Structural neuroimaging should be used in all patients with suspected dementia, preferably using MRI* (Level A)*. In the impossibility of using MRI, CT can serve as an alternative method *(Level B).* Hippocampal atrophy must be assessed in all patients with the aim of reaching a diagnosis of pure VaD or VaD associated to AD *(Level B).* Neurovascular assessment can be necessary for clinical clarification and determining therapeutical interventions (*Good Practice Point*).

### Functional neuroimaging

Functional aspects of neuroimaging often provide useful supplementary information to the diagnosis, such as proton MR spectroscopy (^1^H MRS), CT and MRI perfusion, besides isotopic techniques including Single Photon Emission Computed Tomography and Positron Emission Tomography (PET).

**Proton spectroscopy** - ^1^H MRS represents biochemical information via MRI, which can be obtained at the same time as structural acquisitions. Studies in AD have shown changes in mI/Cr in the posterior region of the cingulate (PC)^103^ and reduced Naa/Cr in hippocampi (HCs), with progressive decline according to stage of the disease.^104^ In addition, comparing alterations in HCs with those in PC for AD enables staging by spectroscopy.^105^

Studies of these structures in VaD (HCs, PC) are scarce. Investigations have been performed mainly for PC, comparing findings in several different dementia types (AD, FTLD, DLB) and subcortical VaD in mild stages, involving a small number of patients, and have revealed reduced Naa/Cr, although within one standard deviation of the normal group.^6^ Other studies however, found no significant difference in Naa/Cr ratio of PC between AD and VaD.^107^ A study focusing on HCs (and frontal and parietal lobes) in dementia without vascular lesions (AD) and with vascular lesions (lacunes)(subcortical VaD) showed a lower Naa/Cr ratio in HCs among AD cases, whereas this ratio was similar in dementia patients with lacunes and in normal controls.^108^ ^1^H MRS can be used as a resource in diagnosing VaD, particularly as a differential item with AD and in suspected cases of AD+VaD (MD).^6^

**Isotopic neuroimaging** - SPECT and PET are used fairly frequently in diagnosing dementia. Some studies using SPEC were designed to compare AD with other types of dementia and have shown sensitivity and specificity for AD vs. VaD of 71% and 75%, respectively.^109^ However, although results were variable, the techniques seems to be useful for distinguishing AD from VaD, but use for diagnostic purposes without previous structural imaging is not advised.^110^ PET can differentiate AD from VaD by disclosing temporo-parietal pattern of hypometabolism in AD, or predominant frontal lobe damage in VaD.^111^ Different regional patterns of hypoperfusion as seen by SPECT, or hypometabolism seen on PET, can assist in differentiating diverse neurodegenerative types and VaD. Images on SPECT and PET in VaD show diverse patterns according to VaD subtype - such as multifocal pattern seen in dementia due to multiple infarcts, or with a more diffuse pattern associated to extensive lesion of white matter and lacunes.^57^

**Recommendations** - The use of ^1^H MRS can be valuable for certain cases of VaD and for differentiating with MD (*Practice option*). SPECT and PET can be used in cases where there is diagnostic doubt after clinical workup and structural imaging, but should not be used as a standalone imaging assessment *(Good Practice Point).*

### Blood exam

The blood test is a necessary part of the assessment in cases of cognitive disorder so as to:

(i) identify comorbidities and/or complications;
(ii) reveal potential risk factors;
(iii) explore causes of frequently associated confusional states, and
(iv) less often, identify the primary cause of the dementia. Cognitive disorders can be associated to a broad array of metabolic, infectious, and toxic conditions which must be identified and treated.^30^

Besides routine items (full blood count, ESR, electrolytes, glucose, renal and hepatic function tests, and TSH), items should be ordered that represent vascular risk factors (VRFs) which merit separate consideration in VCI/VaD. VRFs are intrinsically linked to CVD (and transient vascular ictus or report of ictus symptoms in its absence).^112^ These are also associated to reports of ictus symptoms in the absence of diagnosed ictus or transient ischemic attack.^113,114^ VRFs are numerous and encompass metabolic, toxic, genetic, cardiovascular, and demographic factors, being divided into non-modifiable and modifiable (the majority) with this latter group being susceptible to preventive measures.^12,31^ A comprehensive study showed that among the numerous VRFs for cerebral vascular ictus, just 10 were associated to 90% of cases, namely: arterial hypertension, smoking, waist-hip ratio, high risk diet, moderate physical activity, diabetes mellitus, excessive alcohol intake, psychosocial stress and depression, cardiac causes and ratio of apolipoproteins B for A1 (Class I).^115^ It has been suggested that ≥3 VRFs among those cited place the brain at high risk of cognitive impairment.^30^ Cognitive decline associated to VRFs in the absence of clinical ictus of dementia has also been observed, with theory proposing that subclinical CVD proves an important link among the main VRFs for ictus and cognitive function.^117^ Healthy elderly can present subclinical CVD,^118^ leading to the hypothesis that CVD, faster cerebral atrophy, abnormal cerebral white matter and clinically asymptomatic brain infarcts represent possible mechanism linking VRFs to risk of future ictus and cognitive dysfunction.^119^ Genetic risk factors are examined separately (see "Genetic exam").

**Recommendations** - Blood tests should be performed at first assessment, including routine items and those representing a potential cause of cognitive impairment or as comorbidity, including exams which represent VRFs (*Level A*). More in-depth tests can be necessary in selected cases (*Good Practice Point*).

### Other exams

This category includes exams performed in special situations according to specific indications.

**Cerebrospinal fluid** - The cerebrospinal fluid exam (CSF) has an important place in diagnosing neurodegenerative dementia (AD, FTD, DLB, CJD) through the study of markers based on β-amyloid peptide (βA) and total tau and phospho-tau protein.^54^ Levels of Aβ42 are reduced in AD (and in AD+CVD) and increased in VaD, enabling AD (and AD+CVD) to be discriminated from VaD. Aβ42 has proved an important resource for discriminating AD vs. VaD and possibly improving the diagnostic precision of cases classified as MD (presence of CVD on neuroimaging). Tau protein is high in AD (and in AD+CVD) and low in VaD.^120-122^ Levels of phospho-tau exhibit differentiated increase, being greater in AD, intermediate in AD+CVD and lower in VaD.^122,123^ Analysis correlating Aβ42 and total tau have shown high specificity in AD and low specificity in VaD 48% [29-67]), possibly owing to the presence of neurodegenerative lesion in this condition.^120^

**Recommendations** - The CSF exam is recommended in certain situations (inflammatory diseases, vasculitis, rapidly progressive dementia)(*Good Practice Point*). The markers based on tau protein and Aβ42 can be used as a complement in cases with diagnostic doubt (*Level B*).

**Electroencephalograph** - EEG in cases of AD, AD+ CVD (with pathological confirmation) and controls discloses abnormalities in the majority of patients from pathological groups, whereby normal EEG has shown a negative predictive value of 0.825 for AD diagnosis (Class II).^124^ Visual EEG and quantitative EEG (qEEG) can be used in the differential diagnosis between AD and subcortical VaD.^125^ EEG can be required under some circumstances, such as in cases of diagnostic doubt,^126^ but does not constitute a routine exam in dementia.^127^

**Recommendations** - The EEG can be a useful complement in the diagnostic process (*Practice Option*). It can be used in the differential diagnosis of transient epileptic amnesia vs. transient ischemic attack (*Level B*).

**Genetic exam** - Vascular ictus of any etiology, whether familial or genetic, is a basic cause of VaD (and of vascular CIND).^128-130^ A positive family history seems to constitute a risk factor for ictus and genetic influence can vary according to ictus subtype.^131,132^ There is a strong relationship between familiar conditions associated to ischemic and hemorrhagic stroke, as well as those related to connective tissue disease and hematologic diseases, among others.^128-130^ Single gene causes of stroke are considered relatively rare with multiple genetic influences on VRFs more common, influencing pathogenesis and severity.^129^ The monogenic disorders associated to CVD include CADASIL (*NOTCH 3*), hereditary variant of cerebral amyloid angiopathy (CAA), the frequent sickle cell disease (HBB*S [homo and heterozygotes] and haplotypes β^*S*^), Fabry's disease (GLA), homocystinuria (CBS and other genes), besides other rare conditions.^26,64,133-136^

**Recommendations** - The genetic exam for detection of known pathogenic mutations can be carried out when available, mainly for genetic counselling and clinical research purposes. The exams should be done in a specialized center, with appropriate counselling of patient and family members (*Good Practice Point*).

Certain investigations can yield important information for diagnosis (such as concentrations of enzymes, aminoacids, antibodies among others). The biopsy of tissues such as the skin test in the CADASIL, as well as the brain, can be important in primary vasculitis.^137,138^

**Recommendations** - Specific exams and tissue biopsy can offer a specific diagnosis in some rarer conditions. The exam should be carried in specialized centers in carefully selected cases (*Good Practice Point*).

## Conclusion

The assessment procedures for diagnosing VaD require multi-disciplinary interaction toward reaching a diagnosis. This part of the proposal addressed the analysis of diagnostic criteria, anamnesis, as well as clinical and supplementary exams (neuroimaging and laboratory) used for diagnosing VaD, classified according to proven evidence at various levels.

It should be highlighted that only around half of the population of patients with VCI/VaD present with dementia and the group envisages that the present study can be further refined to enable more precise diagnosis of this condition and that part of the spectrum of CIND and vascular MCI can be extended with the defining of suitable criteria and diagnostic process.

## GROUP RECOMMENDATIONS IN ALZHEIMER'S DISEASE AND VASCULAR DEMENTIA OF THE BRAZILIAN ACADEMY OF NEUROLOGY

**Amauri B. da Silva** [UNINEURO, Recife (PE)]; **Ana Cláudia Ferraz** [Serviço de Neurologia do Hospital Santa Marcelina (SP)]; **Analuiza Camozzato de Pádua** [Universidade Federal de Ciências da Saúde de Porto Alegre (UFCSPA); Hospital de Clínicas de Porto Alegre (UFRGS) (RS)]; **Antonio Lúcio Teixeira** [Departamento de Clínica Médica, Faculdade de Medicina da Universidade Federal de Minas Gerais, Belo Horizonte (MG)]; **Ayrton Roberto Massaro** [Instituto de Reabilitação Lucy Montoro (SP)]; **Benito Pereira Damasceno** [Departamento de Neurologia da Universidade Estadual de Campinas (SP)]; **Carlos Alberto Buchpiguel** [Departamento de Radiologia, Faculdade de Medicina da Universidade de São Paulo (SP)]; **Cássio Machado C. Bottino** [Programa Terceira Idade, Instituto de Psiquiatria do Hospital das Clínicas da Faculdade de Medicina da Universidade de São Paulo (FMUSP) (SP)]; **Cláudia C. Godinho** [Serviço de Neurologia do Hospital de Clínicas de Porto Alegre, Universidade Federal do Rio Grande do Sul (RS)]; **Cláudia Sellitto Porto** [Grupo de Neurologia Cognitiva e do Comportamento da Faculdade de Medicina da USP (SP)]; **Delson José da Silva** [Núcleo de Neurociências do Hospital das Clínicas da Universidade Federal de Goiás (UFG); Instituto Integrado de Neurociências (IINEURO), Goiânia (GO)]; **Elza Dias-Tosta** [Presidente da Academia Brasileira de Neurologia, Hospital de Base do Distrito Federal (DF)]; **Emílio Herrera Junior** [Departamento de Medicina Interna, Faculdade de Medicina de Catanduva (SP)]; **Francisco de Assis Carvalho do Vale** [Universidade Federal de São Carlos (UFSCar), Departamento de Medicina (DMed) (SP)]; **Gabriel R. de Freitas** [Instituto D'or de Pesquisa e Ensino; Universidade Federal Fluminense (RJ)]; **Hae Won Lee** [Instituto de Radiologia, Hospital das Clínicas da Faculdade de Medicina da Universidade de São Paulo e Hospital Sírio-Libanês (SP)]; **Jerusa Smid** [Grupo de Neurologia Cognitiva e do Comportamento do Hospital das Clínicas da Faculdade de Medicina da Universidade de São Paulo (FMUSP) (SP)]; **João Carlos Barbosa Machado** [Aurus IEPE - Instituto de Ensino e Pesquisa do Envelhecimento de Belo Horizonte; Faculdade de Ciências Médicas de Minas Gerais (FCMMG), Serviço de Medicina Geriátrica do Hospital Mater Dei (MG)]; **José Antonio Livramento** [Laboratório de Investigação Médica (LIM) 15, Faculdade de Medicina da Universidade de São Paulo (SP)]; **Letícia Lessa Mansur** [Grupo de Neurologia Cognitiva e do Comportamento do Departamento de Neurologia da FMUSP; Departamento de Fisioterapia, Fonoaudiologia e Terapia Ocupacional da Faculdade de Medicina da USP (SP)]; **Márcia Lorena Fagundes Chaves** [Serviço de Neurologia do Hospital de Clínicas de Porto Alegre, Universidade Federal do Rio Grande do Sul (RS)]; **Márcia Radanovic** [Laboratório de Neurociências - LIM27, Departamento e Instituto de Psiquiatria da Faculdade de Medicina da Universidade de São Paulo (FMUSP) (SP)]; **Márcio Luiz Figueredo Balthazar** [Universidade Estadual de Campinas (UNICAMP), Faculdade de Ciências Médicas (FCM), Departamento de Neurologia (SP)]; **Maria Teresa Carthery-Goulart** [Grupo de Neurologia Cognitiva e do Comportamento do Departamento de Neurologia da Faculdade de Medicina da USP; Centro de Matemática, Computação e Cognição, Universidade Federal do ABC (SP)]; **Mônica S. Yassuda** [Grupo de Neurologia Cognitiva e do Comportamento do Departamento de Neurologia da Faculdade de Medicina da USP; Departamento de Gerontologia, Escola de Artes, Ciências e Humanidades da USP (EACH/USP Leste) (SP)]; **Nasser Allam** [Universidade de Brasília (UnB), Laboratório de Neurociências e Comportamento, Brasília (DF)]; **Norberto Anizio Ferreira Frota** [Universidade de Fortaleza (UNIFOR), Serviço de Neurologia do Hospital Geral de Fortaleza (HGF) (CE)]; **Orestes Forlenza** [Laboratório de Neurociências - LIM27, Departamento e Instituto de Psiquiatria da Faculdade de Medicina da Universidade de São Paulo (FMUSP) (SP)]; **Paulo Caramelli** [Departamento de Clínica Médica, Faculdade de Medicina da Universidade Federal de Minas Gerais, Belo Horizonte (MG)]; **Paulo Henrique Ferreira Bertolucci ** [Universidade Federal de São Paulo (UNIFESP), Setor de Neurologia do Comportamento - Escola Paulista de Medicina, São Paulo (SP)]; **Regina Miksian Magaldi** [Serviço de Geriatria do Hospital das Clínicas da FMUSP, Centro de Referencia em Distúrbios Cognitivos (CEREDIC) da FMUSP (SP)]; **Renata Areza-Fegyveres** [Grupo de Neurologia Cognitiva e do Comportamento do Hospital das Clínicas da Faculdade de Medicina da Universidade de São Paulo (FMUSP) (SP)]; **Renato Anghinah** [Grupo de Neurologia Cognitiva e do Comportamento do Hospital das Clínicas da Faculdade de Medicina da Universidade de São Paulo (FMUSP); Centro de Referência em Distúrbios Cognitivos (CEREDIC) da FMUSP (SP)]; **Ricardo Nitrini** [Grupo de Neurologia Cognitiva e do Comportamento do Hospital das Clínicas da Faculdade de Medicina da Universidade de São Paulo (FMUSP); Centro de Referência em Distúrbios Cognitivos (CEREDIC) da FMUSP (SP)]; **Rodrigo Rizek Schultz** [Setor de Neurologia do Comportamento do Departamento de Neurologia e Neurocirurgia da Universidade Federal de São Paulo, Núcleo de Envelhecimento Cerebral (NUDEC) - Instituto da Memória (UNIFESP) (SP)]; **Rogério Beato** [Grupo de Pesquisa em Neurologia Cognitiva e do Comportamento, Departamento de Medicina Interna, Faculdade de Medicina, UFMG (MG)]; **Sonia Maria Dozzi Brucki** [Grupo de Neurologia Cognitiva e do Comportamento da Faculdade de Medicina da Universidade de São Paulo; Centro de Referência em Distúrbios Cognitivos (CEREDIC) da FMUSP; Hospital Santa Marcelina (SP)]; **Tânia Novaretti** [Faculdade de Filosofia e Ciências, Campus de Marília, da Universidade Estadual Paulista (UNESP) (SP)]; **Valéria Santoro Bahia** [Grupo de Neurologia Cognitiva e do Comportamento do Hospital das Clínicas da Faculdade de Medicina da Universidade de São Paulo (FMUSP) (SP)]; **Ylmar Corrêa Neto** [Universidade Federal de Santa Catarina (UFSC), Departamento de Clínica Médica, Florianópolis (SC)].
